# The (in)visibility of fibromyalgia through its symptoms and the challenges of its diagnosis and therapy

**DOI:** 10.1590/0034-7167-2023-0363

**Published:** 2024-06-14

**Authors:** Larissa Pereira Costa, Márcia de Assunção Ferreira

**Affiliations:** I Universidade Federal do Rio de Janeiro. Rio de Janeiro, Rio de Janeiro, Brazil

**Keywords:** Fibromyalgia, Diagnosis, Therapeutics, Nursing, Social Psychology, Fibromialgia, Diagnóstico, Terapéutica, Enfermería, Representación Social

## Abstract

**Objective:**

To analyze the social representations of fibromyalgia based on its symptoms and their influences on diagnosis and therapy.

**Methods:**

Qualitative research with the application of the Theory of Social Representations and snowball sampling method. Semi-structured interviews were conducted with 30 adults diagnosed with fibromyalgia in the city of Rio de Janeiro, Brazil, between April 2020 and January 2021. Statistical and lexicographical analysis was performed using Alceste software.

**Results:**

Pain, as a subjective phenomenon, complicates its legitimacy, diagnosis, and therapy, enhancing suffering. Insufficient information generates judgments, stereotypes, and prejudices.

**Final Considerations:**

Stigmas, prejudices, the variety and invisibility of symptoms make it difficult to objectify the disease within the Cartesian-biomedical frameworks, generating diagnostic pilgrimage, mistakes, and challenges in treatment. Such representations hinder relationships and the management of the disease. Deconstructing them is a way to better care for those with fibromyalgia. Raising awareness and spreading qualified information are important allies.

## RESUMEN

**Objetivo:**

Analizar las representaciones sociales de la fibromialgia basadas en sus síntomas y sus influencias en el diagnóstico y la terapia.

**Métodos:**

Investigación cualitativa con la aplicación de la Teoría de las Representaciones Sociales y método de muestreo en bola de nieve. Se realizaron entrevistas semiestructuradas con 30 personas adultas, diagnosticadas con fibromialgia, en la ciudad de Río de Janeiro, Brasil, entre abril de 2020 y enero de 2021. Se llevó a cabo un análisis estadístico y lexicográfico mediante el software Alceste.

**Resultados:**

El dolor, como fenómeno subjetivo, dificulta su legitimidad, el diagnóstico y la terapia, intensificando el sufrimiento. La información insuficiente genera juicios, estereotipos y prejuicios.

**Consideraciones finales:**

Los estigmas, prejuicios, la variedad y la invisibilidad sintomática dificultan la objetivación de la enfermedad en los modelos cartesianos-biomédicos, generando una peregrinación diagnóstica, errores y desafíos en el tratamiento. Tales representaciones obstaculizan las relaciones y el manejo de la enfermedad. Desconstruirlas es un camino para mejorar el cuidado de quienes tienen fibromialgia. Sensibilizar a las personas y difundir información cualificada son aliados importantes.

## INTRODUCTION

Fibromyalgia is a chronic rheumatic condition characterized by widespread pain lasting more than three months, fatigue, cognitive and functional changes, sleep disturbances, and viscerointestinal dysfunctions, and it is commonly associated with anxiety and depression^(1, 2, 3)^. Its etiopathogenesis is complex and not yet fully understood, as is its prevalence in females, with a ratio of up to nine women, typically in the age group of 35 to 44 years, for every man^(4, 5)^. A review study emphasizes the prevalence of fibromyalgia among females and suggests that this might be a risk factor for the condition^(6)^.

Fibromyalgia affects 4.2% of women aged between 40 and 50 compared to 0.2% of men, with these women typically having lower educational levels and living in conditions of social, political, and economic vulnerability^(5, 7, 8)^. Additionally, women younger than 60.6 years tend to exhibit more severe symptoms of the disease^(7)^. Women are 1.5 times more likely to experience widespread chronic pain than men and are ten times more likely to have 11 or more tender points during clinical examination, which may explain the higher prevalence of fibromyalgia in women^(9)^.

Worldwide, the incidence of fibromyalgia ranges from 0.2% to 8%^(3)^. It is estimated to affect 2% to 4% of the global population and up to 2.5% of the Brazilian population^(4, 5)^. However, due to significant symptomatic heterogeneity and the lack of standardized diagnostic procedures, these figures may vary and influence the approach to treatment^(10)^.

The diagnosis of fibromyalgia is primarily clinical and dependent on the examiner, based on evaluating pain and sensitivity at tender points, combined with the clinical judgment of the signs and symptoms described by patients. This accounts for the numerous diagnostic variations depending on the physician’s experience, as there is no laboratory marker or imaging test to confirm its presence^(4, 9)^.

In an attempt to reduce these diagnostic disparities, the American College of Rheumatology (ACR) established criteria in 1990 to guide the clinical and diagnostic assessment of individuals with fibromyalgia. However, these criteria overly emphasized the identification of tender points at the expense of other symptoms. In 2010, the ACR revised these criteria to underscore the importance of clinical history, assessment of widespread pain, and symptom severity for diagnosis. In 2017, a document with new guidelines was issued, in which experts recommended applying the 2010 diagnostic criteria and advised evaluating the tender points along with other presented functional disorders, such as diffuse pain, fatigue, sleep disturbances, and cognitive changes^(4, 11)^. This development highlights the degree of diagnostic disagreements and the magnitude of this challenge given the symptoms’ non-specificity and subjectivity.

Therefore, research suggests there may be overdiagnosis of fibromyalgia in women and underdiagnosis in men, leading to inaccuracies in statistics related to symptoms, prevalence, costs, comorbidities, and clinical outcomes. Nevertheless, these studies also propose that there is a significant element of social construction surrounding fibromyalgia and its identification, underscoring the medical and social aspects of the condition^(12, 13)^.

This extensive variation in the clinical manifestations of fibromyalgia is influenced by social, psychological, and cultural aspects^(14)^. Therefore, it can be stated that fibromyalgia, in addition to its biological and somatic variables, also involves psychosocial aspects that permeate the entire health/disease process, negatively affecting the physical, cognitive, social, familial, and professional aspects of those afflicted^(7)^.

In this context, a question arises: How are the symptoms of fibromyalgia expressed in the social representations of people with this disease, and what impact do these have on its diagnosis and treatment?

The motivation for exploring this research question is that individuals with fibromyalgia perceive the condition as a stigmatized, invisible, and difficult-to-understand disorder^(15)^, which exacerbates the suffering of those living with this illness, eliciting human responses that could be more effectively managed by nursing once they are understood.

Psychosocial aspects related to health and disease phenomena indicate an intersection between psychology and sociology in their comprehension, and an effective tool for studying such phenomena is through social representations^(16, 17)^. Social representations are forms of practical knowledge that create connections between understanding and action, shaped by the process of making what is abstract and unfamiliar tangible, known as objectification, and by integrating this with existing reference frameworks for what is already known or familiar, referred to as anchoring. Therefore, understanding social representations entails uncovering their formation processes when employing a socio-anthropological approach^(17)^.

## OBJECTIVE

To analyze the social representations of fibromyalgia based on its symptoms and their influences on diagnosis and treatment.

## METHODS

### Ethical aspects

The project was submitted to the Research Ethics Committee of the Anna Nery School of Nursing and the São Francisco de Assis Health Institute at Universidade Federal do Rio de Janeiro and was approved on March 16, 2020. At the time of the invitation to participate in the study, the principal investigator introduced herself to the contacted individuals, informed them about the research, and all participants signed an informed consent form upon accepting the invitation.

### Theoretical and Methodological Framework

The Theory of Social Representations framework was applied, considering the concepts of objectification and anchoring in the formation of social representations^(16, 17)^.

### Type of Study

This is a qualitative, descriptive, and exploratory study, applying the Consolidated Criteria for Reporting Qualitative Research (COREQ) guidelines.

### Methodological Procedures

Participants were recruited using the snowball sampling method, employed when there are difficulties accessing the target population of the study or when initial probabilistic sampling is impractical^(18)^. Recruitment began with two “seeds,” women diagnosed with fibromyalgia residing in the state of Rio de Janeiro, referred by someone from the principal researcher’s network. Through these two referrals (seeds), new participants fitting the study profile were recruited, all contacted by telephone. Data collection was concluded after reaching 30 participants, based on the consensus recommendation for qualitative samples and the criterion of data sufficiency saturation^(19)^. All contacted individuals agreed to participate in the study, and there were no dropouts.

### Data Source

Inclusion criteria for participants were being 18 years or older and having a diagnosis of fibromyalgia. Exclusion criteria were individuals with cognitive or speech impairments that would hinder data collection, and adolescents or young people diagnosed with Juvenile Fibromyalgia Syndrome, as this condition requires a different analysis of the disease’s impact on daily life.

### Data Collection and Organization

The principal researcher conducted the data collection from April 2020 to January 2021, through individual face-to-face interviews with voice recording, lasting between 60 and 90 minutes. Current safety protocols during social distancing due to COVID-19 were adhered to. Interviews were conducted at the participants’ homes, at their discretion.

Two instruments were utilized: the first consisted of questions for collecting personal and occupational data (such as gender, age, marital status, occupation), health-disease history, family history, clinical and medication history, symptoms, health care follow-up (private or public network), use of non-pharmacological treatment methods, participation in support groups, and frequency. The second instrument comprised open-ended questions, presented through a semi-structured question guide, exploring who knows, from where knows, what and how knows about the phenomenon, its effects, and impacts^(17)^. Essentially, it involved exploring knowledge and practices regarding the researched subject. Accordingly, the questions addressed the disease and its clinical manifestations, discovery, diagnosis and treatment challenges, relationships with individuals with and without the disease, care practices and learnings, challenges, support networks, and areas needing further understanding regarding the disease and care.

### Data Analysis

The participant profile data were statistically analyzed, applying both absolute and relative frequencies. The interview texts formed a corpus of 30 initial context units (ICUs) after transcription, corresponding to the number of interviews processed by the Alceste software. This software conducts a contextual lexical analysis of a collection of text segments and performs calculations on the co-occurrence of words within these segments to define word classes that represent different discourses concerning the research object. In doing so, it identifies lexical oppositions and uncovers the contrasts between different collective viewpoints expressed in the text’s vocabulary, enabling access to intergroup communication, knowledge sharing, and the generation of social representations^(20)^.

The text processing within the software facilitates the grouping of semantic roots based on the words used by participants in their discourses to communicate their perspectives on the subject under investigation. From this grouping, the software generates figures (dendrograms), such as the Descending Hierarchical Classification (DHC), which emphasizes the most significant words of the class through statistical association, expressed by the Phi value. The DHC is comprised of the words’ reduced forms based on their roots, and the complete forms (the words) are listed in the body of the report generated by the software, along with their respective frequencies of appearance in lexical classes and the statistical value of association with the class (Phi value). To understand the reasons behind the occurrence of certain words, the text fragments constituting each class are examined, expressing the concepts that lend them significance, referred to as Elementary Context Units (ECUs).

To achieve the set objective, this article focuses on the two classes derived from the lexicons that deal with the symptoms, diagnostic journey, and therapy of fibromyalgia. Class 2, titled “A wandering pain: fibromyalgia and its symptoms,” and Class 3, titled “Diagnostic challenges: a path of pilgrimage,” are discussed as these classes pertain to the extensive symptomatology of the disease and the diagnostic challenges arising from its lack of specificity.

## RESULTS

Regarding the profile of the 30 participants, there was a predominance of females at 93% (n=28). In terms of age groups, 67% are between 41 to 60 years old (n=20), followed by 30% aged between 20 to 40 years (n=9), and 3% are 61 years or older (n=1). Additionally, 63% are married (n=19) and 70% are employed (n=21). Regarding the duration since the fibromyalgia diagnosis, 40% were diagnosed 10 years ago or more (n=12). Health monitoring is primarily conducted through the private healthcare network by 93% of the participants (n=28). As for treatment, 97% (n=29) use pharmaceuticals, and 3% (n=1) do not; 53% do not follow non-pharmacological treatments (n=16), while 47% use some form of non-pharmacological treatment (n=14). Participation in support groups is reported by 40% (n=12), all of whom participate in virtual groups on social networks such as Facebook, WhatsApp, and YouTube channels.

Regarding the interview data, the software subdivided the text into 4,034 ECUs, composed of 8,138 distinct forms. The software reduced these words to distinct roots, resulting in 1,287 analyzable words and 309 supplementary words. A total of 2,934 ECUs were analyzed, resulting in 73% utilization of the corpus.

Class 2 consisted of 370 ECUs and 112 analyzable words, accounting for 13% of the classified corpus for analysis. In analyzing the Descending Hierarchical Classification (DHC) of this class, the root “Pern” had the highest Phi value, corresponding to 0.32. This root is manifested in the complete forms “*perna*” (leg) and “*pernas*” (legs). Following are the terms “*sono*” (sleep), “*dorm*” (relating to sleep or sleeping), “*dor*” (pain), and “*doi*” (hurts), as shown in Chart 1.

**Chart 1:** Representative words of Class 2, according to the dendrogram of the Descending Hierarchical Classification (DHC), Rio de Janeiro, Brazil, 2021

CLASS 2 – 370 ECU		
Reduced form (in portuguese)	Complete Form	Phi Value
*pern*	Leg (35), legs (47)	0.32
*sono*	Sleep (48)	0.26
*dorm*	Sleeps (5), numbness (6), numb (6), was sleeping (5), to sleep (51), slept (1)	0.23
*dor*	Pain (295), pains (68)	0.23
*doi*	Hurts (49), was hurting (15)	0.22
*nas*	In the (64)	0.21
*durmo*	I sleep (24)	0.19
*braço*	Arm (12), arms (17)	0.17
*joelho*	Knee (28), knees (4)	0.17
*acord*	Wakes up (2), woken (3), to wake up (3), was waking (9), woke up (4), I wake up (13)	0.16
*pescoço*	Neck (15)	0.16
*cost*	Back (22), rib (3), ribs (2)	0.16
*noite*	Night (14), nights (2)	0.15
*insônia*	Insomnia (15)	0.15
*sensibilida*	Sensitivity (13)	0.15
*and*	Walks (7), walking (4), to walk (18), was walking (5), I walked (4), I walk (3)	0.14
*sint*	I feel (78)	0.13
*amanhec*	Dawns (2), dawning (1), was dawning (3), I dawn (4)	0.13
*musculatu*	Musculature (9)	0.13
*mão*	Hand (13), hands (16)	0.13
*parec*	Seems (34), seemed (12)	0.12
*forte*	Strong (16), stronger (7)	0.12
*inch*	Swells (2), swelling (1), swollen (female) (2), swollen (male) (2), swelling (action) (4), to swell (1), was swelling (1)	0.12
*semana*	Week (26), weeks (2)	0.12
*muscul*	Muscles (6)	0.11
*pé*	Foot (24)	0.11
*cansaço*	Tiredness (17)	0.11

Named “A Wandering Pain: Fibromyalgia and Its Symptoms,” Class 2 highlights the lexicons associated with the symptomatic presentation of the disease and how, when, and where these individuals feel these discomforts. The results indicate an attempt to identify the symptoms presented by the disease, as well as their location, duration, and intensity, as an observational strategy to understand how the disease manifests, how it is perceived, characterized, and which situations and behaviors intensify or minimize it.

*Pain, a lot of pain, the joints hurt more in the upper part, finger, all my joints, arms, and back. Lately, I hadn’t felt much pain in my legs, but now I’ve been feeling it a lot.* (ECU 24, woman, 41-60 years)

*The symptoms are pain and fatigue. Discouragement, you never rest. And the body. It’s as if you never really had any rest. It feels like you’re doing very heavy, manual labor twenty-four hours a day. So, the doctor told me that issues with sleep, mood, everything would be related to fibromyalgia.* (ECU 9, woman, 41-60 years)

*It’s different from me carrying a heavy backpack, it’s different, the weight will be in one specific place, on that day. It will be in my legs, so I will have unbearable pain in my legs, or my neck. How many times have I cried asking God to take away my neck, take away my legs, because it’s unbearable pain when I’m having a flare-up.* (ECU 6, woman, 20-40 years)

*That’s why I call it that, it’s a wandering pain, it’s not a permanent pain in one place. And when the flare-up happens, it hurts, and it hurts a lot. I went through a phase where I had pain in this little finger here. Anything I held in my hand, I would drop. I had to get up to grab things, and it hurt, I would even get shocks.* (ECU 10, woman, 41-60 years)

*So, initially, I had pain in my arm, but after two days, that pain was in my chest and neck. And then I had to observe how this happened, how long it took for the pain to move to another part of the body, where it hurt the most.* (ECU 17, woman, 20-40 years)

*Because I feel pain constantly, you know?! I’ve noticed, the joints hurt a lot, but I’ve also observed that when I get nervous for some reason, or anxious about something, the pain comes on intense, it’s more intense, you know?!* (ECU 19, woman, 41-60 years)

Class 3, in turn, was comprised of 637 ECUs and 129 analyzable words, representing 22% of the corpus classified for analysis. Upon analyzing the DHC of this class, the term “*Exame*” exhibited the highest Phi value, corresponding to 0.32. This root is represented in the complete forms “*exame*” (exam) and “*exames*” (exams). Following this, the terms “*reumatolog*” (rheumatologist), “*tom*” (take), “*med*” (medication), and “*remed*” (remedy) are noted, as shown in Chart 2.

**Chart 2:** Representative words of Class 3, according to the dendrogram from the Descending Hierarchical Classification (DHC), Rio de Janeiro, Brazil, 2021

CLASS 3 – 637 ECU		
Reduced Form (In portuguese)	Complete Form	Phi Value
*exame*	Exam (62), exams (80)	0.32
*reumatolog*	Rheumatological (1), rheumatological (feminine) (1), rheumatological (masculine) (2), rheumatologist (89)	0.27
*tom*	Take (15), taken (5), taking (47), to take (72), would take (2), was taking (19), take (imperative) (1)	0.26
*med*	Measure (1), measured (2), medical (feminine) (15), to medicate (1), doctor (153), doctors (21), to measure (3)	0.23
*remed*	Little remedy (1), remedy (82), remedies (31)	0.21
*medica*	Medication (60), medications (28)	0.20
*ortoped*	Orthopedics (3), orthopedic (feminine) (1), orthopedist (37), orthopedists (5)	0.20
*consult*	Consultation (19), to consult (9), consultations (3), was consulting (1), consulted (6), I consult (1)	0.18
*fiz*	Did (79), would do (3), did (plural) (6), would do (subjunctive) (5)	0.17
*psiquiatr*	Psychiatrist (49), psychiatric (1)	0.17
*os*	The (150)	0.16
*sangue*	Blood (26)	0.16
*velija*	Lyrica (32)	0.16
*tratament*	Treatment (60), treatments (4)	0.16
*doutor*	Doctor (26), doctor (feminine) (13)	0.15
*itaperuna*	Itaperuna (27)	0.15
*ano*	Year (43), years (108)	0.15
*diagnostic*	Diagnosed (feminine) (7), diagnosed (masculine) (4), to diagnose (5), diagnosed (plural) (2)	0.14
*neurolog*	Neurological (1), neurologist (24), neurologists (1)	0.14
*fiqu*	Stay (1), stayed (72)	0.14
*mes*	Month (12), table (1), months (37)	0.13
*uns*	Some (81)	0.13
*marc*	Mark (3), marked (2), to mark (10), I mark (8), marked (3)	0.13
*fez*	Did (50)	0.13
*trat*	Treats (2), treated (1), treating (13), to treat (22), treated (plural) (2), was treating (2), treated (1st person) (6)	0.13
*nel*	In her (3), in him (24)	0.13
*intern*	Admit (1), admitted (feminine) (4), admitted (masculine) (6), to admit (6), admitted (plural) (1), would admit (1)	0.13

This class is closely associated with the diagnostic process of the disease, as well as with the therapeutic approaches linked to it.

Class 3 has been named “Diagnostic Challenges: A Pilgrimage Path.” The ECUs of this class relate to the diagnostic and therapeutic processes of fibromyalgia. Through the lexicons, the journey to identify the source of the pain is understood, encompassing its chronological aspects and comprehension, as well as methods capable of alleviating it. The findings indicate that individuals with fibromyalgia embark on a prolonged quest for a diagnosis, frequently leading them to question whether they actually suffer from a disease, thus casting doubt on their sensations and the intensity thereof.

As fibromyalgia is a clinical diagnosis, it often becomes a diagnosis of exclusion, owing to the lack of tests and laboratory markers for its confirmation. Additionally, the condition is frequently linked with other diagnoses, which may lead to an increase in underdiagnoses. This extensive search not only magnifies the symptoms these individuals experience but also amplifies depressive and anxious states.

*And I was hospitalized for ten days. They performed all kinds of tests: CT scans, electrocardiograms, MRIs, blood tests, every possible examination, and nothing showed up, right?! Then, I went from the hospital to a neurologist, to a cardiologist. Later, I went to a rheumatologist. It was the rheumatologist who figured it out.* (ECU 20, male, 20-40 years)

*I started taking the medication Lyrica and improved a lot, a lot, a lot. I went back to him, he reviewed the tests and said: indeed, you don’t have rheumatoid arthritis. Your diagnosis is fibromyalgia. And then it was a liberation because then you escape from that madness, what you have, why you don’t get better.* (ECU 21, woman, 41-60 years)

*And that’s when I discovered it and started treatment. Oh, about eight months, eight months experiencing pain. In fact, during those eight months, I would go to the emergency room, arrive there in a lot of pain, and they wanted to administer morphine by any means.* (ECU 17, woman, 20-40 years)

*I don’t know what that thing the native sells is. I don’t even know what it is, but what I take is what relieves me. No, it has no name, nothing. It comes like this, ten aluminum foil envelopes, I’ll show you, folded this size, with white powder inside.* (ECU 26, woman, 41-60 years)

*Everyone I’ve met in person, including from here, went to rock bottom with depression. I’ve been there, I’m telling you I didn’t have a panic syndrome or anything like that, but I was, I’ve already spent months, there in the bedroom*. (ECU 5, woman, 41-60 years)

## DISCUSSION

The participant profile reaffirms what is known about the group affected by fibromyalgia, which is predominantly composed of women and adults. However, the scarcity of studies with substantial male samples hinders in-depth and accurate analyses regarding the disease’s specificities in relation to gender^(6)^.

Regarding clinical characteristics, fibromyalgia is known for its wide range of symptoms, as demonstrated by the data from Class 2. However, among the signs and symptoms most reported by those living with this disease, pain is the central symptom, with variations only in its location. This finding aligns with other studies that identify pain as the most expressive, recurrent, and impactful symptom for people with fibromyalgia^(21, 22, 23)^.

It is characterized by the presence of chronic pain lasting more than three months, painful palpation at specific sites known as tender points, and the absence of an inflammatory process. Generally, there is a strong relationship between the number of identified tender points and the levels of stress and depression, often justified by the emotional distress commonly present in those living with chronic pain^(4, 22, 23)^.

Other symptoms frequently mentioned by those with fibromyalgia include non-restorative sleep, excessive fatigue, lack of energy, mood swings, changes in concentration and memory, stiffness and/or muscle weakness, numbness, irritable bowel syndrome, irritable bladder, headache, dizziness, mental confusion, dry mouth, dry eyes, and sensitivity to cold, among others^(24, 25, 26)^.

However, pain is the primary complaint among individuals with fibromyalgia, and it is a very distinct sensory perception. Often, these individuals try to characterize, objectify, and name it to facilitate discussion^(17)^. Among the highlighted characteristics are migrating pain, moving pain, shock-like pain, stabbing pain, burning pain, intense paralyzing pain, nauseating pain, pain that causes hypersensitivity, and cruel and unbearable pain. All these descriptions are attempts to communicate what fibromyalgia is to those who do not experience it firsthand, to objectify something inherently subjective^(16)^. Describing it as “a migrating pain, a pain that moves” is a metaphor that encapsulates the representation of a painful sensation that travels through the body, manifesting in different locations at different times, leading to multiple complaints.

People with fibromyalgia attempt to anchor it in some pre-existing knowledge, an experience, or previously felt pain, characterizing it as an abnormal painful sensation, akin to shock-like, stabbing, and burning sensations. There is a variety of perceptions regarding the painful experience and an effort to characterize it through observation, aiming to understand its behavior, its peculiarities, and how it presents in the individual^(21)^.

Therefore, to characterize it and make it comprehensible to others, people with fibromyalgia realize that observation is essential. Through observation, they discern the similarities and differences in the painful experience under various conditions, such as changes in ambient temperature. They understand the temporality of the pain, which has been present since childhood and carries emotional connotations; the frequency and location of the pain, whether it is constant or intermittent, fixed or moving; and the factors and conditions that either amplify it, such as stress and anxiety, or reduce it, such as the application of pharmacological or non-pharmacological treatments, among other insights.

Therefore, having a central symptom that is subjective and multifactorial, affecting both the somatic and social spheres, understanding fibromyalgia requires careful observation of how the disease and its symptoms behave, how they present themselves, their characteristics, and their impacts on numerous dimensions of life, as well as which care strategies, both pharmacological and non-pharmacological, are effective for its alleviation. The quest to understand it is crucial to guiding the actions to be taken. In this sense, thought, knowledge, and action intersect in the decision-making process on how to respond to the phenomenon^(17)^, in this case, fibromyalgia. This is where the movement of the social representation of fibromyalgia comes into play: developing knowledge about it to learn to talk about one’s condition, manage it, and take action on it^(16)^, in terms of care.

Due to this broad symptomatology and subjectivity, fibromyalgia becomes a significant diagnostic challenge, leading to a true pilgrimage among doctors and specialists in search of a name for the pain, as evidenced by Class 3.

Moreover, it is a syndrome of still unknown cause, labeled as a “medically unexplained” disease because it is not confirmed through traditional tests and diagnostic procedures, but rather by having subjective diagnostic criteria not associated with physical alterations^(24, 27)^. Therefore, until the disease is diagnosed, representations of fibromyalgia depict it as an unexplained and obscure illness, present in both medical and lay discourses, contributing to the dominance of Cartesian biomedical discourse.

People with fibromyalgia report significant difficulties in receiving an accurate diagnosis, a fact that is related to the complexity of its validation and the medical professionals’ lack of knowledge and understanding about the diagnostic criteria and how to evaluate them^(28, 29)^.

Due to its subjective nature, fibromyalgia is often considered an “invisible illness,” and this term also represents a notion that influences the delay in diagnosis and treatment. This invisibility tends to disrupt personal, social, work, and family roles and responsibilities, besides leading to a diagnostic journey that does not always reveal any abnormalities or changes to justify their pain and other symptoms^(27)^. On average, people with fibromyalgia spend between four to ten years waiting for the correct diagnosis^(21,30)^.

This journey in search of a diagnosis, involving numerous medical consultations and tests, generates frustration and discouragement. It is a quest for the legitimization of the pain experienced, where some individuals spend years without knowing exactly what they have and the real diagnosis. This path also involves incorrect treatments, in addition to engendering disbelief and a lack of hope concerning the prognosis.

Therefore, living with fibromyalgia, with all its subjectivity and syndromic nature, results in the experience of stigmatization. Typically, individuals with fibromyalgia are misunderstood regarding their pain, and thus they are rejected and judged as lazy. This stereotyping strengthens and is bolstered by the representations of fibromyalgia as an invisible disease. Representations convey images and ideas about things and people, disseminate information, beliefs, prejudices, judgments, and consequently, shape behaviors and attitudes. The entire stigma of fibromyalgia falls upon the patient, through labels that categorize them into pre-defined images with social effects^(31)^, which is why receiving a diagnosis is seen as a liberation, as noted in ECU 21.

Because it does not present a visible sign, something tangible to justify the pain, the diagnosis of fibromyalgia often becomes predominantly clinical and differential, as well as delayed, dependent on the clinical skills of the evaluating physician^(27, 32, 33)^. This fact underscores the need for investment in medical professional education to apply accurate diagnostic criteria for fibromyalgia, as has been highlighted by studies on the subject^(28, 29)^.

When conducted correctly and comprehensively, while respecting and valuing the patient’s complaint, the diagnostic approach tends to minimize these individuals’ journey through numerous consultations. This reduction in consultations can decrease healthcare costs, the excessive use of pharmacological and non-pharmacological measures without real efficacy, thus preventing complications and hospitalizations^(30, 32)^. Moreover, having knowledge about the disease and understanding that it is real significantly reduces suffering and enhances the prospects for improvement^(32)^.

Regarding therapeutic approaches, pharmacological treatment is widely used for people with fibromyalgia, although there is no specific protocol^(33)^. Conversely, there is low adherence to the continuity of medication treatment, as these medications do not provide a cure and can have undesirable side effects^(1)^. Pharmacological treatments are long-term and often do not lead to effective responses in minimizing pain, in addition to various side effects; for this reason, there is growing interest in adopting non-pharmacological practices complementary to fibromyalgia treatment^(34)^. This interest is supported by participant profile data, showing that the majority (97%) use pharmaceuticals, and nearly half (47%) employ some non-pharmacological method.

Non-pharmacological treatments, such as physical exercise, pilates, physiotherapy, acupuncture, and psychotherapy, are also widely recommended for people with fibromyalgia. Physical exercises in individuals with fibromyalgia reduce pain, improve health-related quality of life, and have a beneficial effect on depression^(35)^. Electrical stimulation and electroacupuncture have also been the subjects of research for fibromyalgia cases, exemplified by a meta-analysis that indicated low-quality evidence for the efficacy of electrical stimulation and moderate-quality evidence for electroacupuncture for pain relief^(36)^. Another non-pharmacological treatment worth mentioning is Integrative Community Therapy, which has proven to be an effective strategy for building and expanding knowledge about fibromyalgia and empowering participants for self-care^(37)^.

Most of the time, non-pharmacological approaches are combined with commonly prescribed medications as alternatives to enhance results in reducing pain and other symptoms. However, not all individuals with fibromyalgia benefit and achieve good results with such therapies. The available therapies will have individualized effects; therefore, each person will experience specific outcomes and determine which therapeutic approach is most suitable for their condition.

However, what is evident is the pursuit of an alternative that provides relief from pain, whatever that alternative may be, its origin and composition, and its possible side effects. The quest for relief leads people to use products about which they do not always know exactly what they are; they adhere to a belief that a particular alternative will produce the desired effect, especially since other therapies have not been satisfactory, as exemplified by the use of “a white powder supplied by a native,” as mentioned in ECU 26.

Another complicating factor for the diagnosis is the association of fibromyalgia with other pathologies. Among the diseases associated with the diagnosis of fibromyalgia, one can mention depression, anxiety, musculoskeletal and joint diseases, autoimmune diseases such as rheumatoid arthritis and psoriatic arthritis, systemic arterial hypertension, among others. The coexistence between fibromyalgia and other pathological conditions can complicate the assessment, diagnosis, and treatment due to an inappropriate interpretation of the clinical presentation of the disease^(30)^.

Thus, the importance of multidisciplinary follow-up and a holistic approach to people with fibromyalgia, beyond the pain, is reaffirmed. The fragmentation proposed by the biomedical model does not satisfactorily meet an individual’s health and complete well-being. There is a greater return to the person’s needs and a higher resolution of their problems when the focus of health care is directed towards their entirety^(38)^.

In this endeavor, understanding the knowledge and practices of care and self-care of people with fibromyalgia can assist healthcare professionals, especially nurses, in seeking care that more frequently meets the needs of those suffering from this condition. Therefore, research on social representations is applied in health, as representations are practical knowledge, whose dimensions are made up of information, attitudes (practices), and the field, which is where the representation organizes itself based on its explanatory elements^(16, 17)^. By uncovering them, professionals can plan health education actions that demystify stereotypes and assist people in making care choices that help them in their treatment processes.

### Study Limitations

This study presents several limitations that should be taken into account when interpreting the results. The first is related to the fact that the research was conducted during the COVID-19 pandemic, which led to the suspension of fibromyalgia support group meetings, and participant recruitment was conducted using the snowball sampling technique. This approach made it challenging to obtain a sample with more diversified characteristics. Consequently, it was not possible to broaden the analyses to facilitate the identification of convergences and divergences in representations by gender and age group.

### Contributions to Nursing, Health, or Public Policy

Understanding the social representations of diseases, particularly those that generate stigma and prejudice, enables nursing professionals to access psychosocial elements that contribute to suffering and influence patient care. Armed with this knowledge, the provision of care can potentially be more precise and tailored to meet the specific needs of patients, as well as to support their families and networks, helping them dismantle representations that hinder relationships and proper management of the disease and the associated suffering. Thus, this understanding enriches the knowledge, care, and strategies for better handling the disease and the patient within their life contexts, through informed insights about the condition.

## FINAL CONSIDERATIONS

Fibromyalgia presents as a syndrome with nonspecific and subjective symptoms, with pain being foremost among them. The diversity and invisibility of its symptoms, lacking a known or apparent cause, impede the disease’s objectification following the Cartesian-biomedical model, reinforce its status as an invisible illness, and lead to various patient descriptions of pain in an effort to characterize and make it tangible for others. Describing fibromyalgia as migrating pain, a “pain that moves,” and thus travels through the body, reflects the cognitive effort of patients to articulate the challenge of pinpointing the disease within the body, as is customary with the biomedical model. This situation results in a diagnostic journey aiming for liberation, a status symbolizing the much-anticipated diagnosis that, until achieved, results in numerous misunderstandings and challenges in the therapeutic management of fibromyalgia. Therefore, enhancing diagnostic accuracy is identified as an urgent requirement in medical training.

Representations are established based on three dimensions: information, attitude, and the context in which the representation is organized based on its explanatory elements. The spread of well-founded information about fibromyalgia serves as an effective means for sensitizing family members and the general public about the disease, facilitating a better understanding, dismantling stereotypes and prejudices, and enabling those afflicted with fibromyalgia to be better understood, accepted, and adequately cared for.
