# Supplementary material for: The (in)visibility of fibromyalgia through its symptoms and the challenges of its diagnosis and therapy
Source: Rev Bras Enferm. 2024 Jun 14;77(2):e20230363. doi: 10.1590/0034-7167-2023-0363 (PMC11178319; doi:10.1590/0034-7167-2023-0363)
Supplement: Translation

# Translation: A (in)visibilidade da fibromialgia por seus sintomas e os desafios do seu diagnóstico e terapêutica

**DOI:** 10.1590/0034-7167-2023-0363pt

**Authors:** Larissa Pereira Costa, Márcia de Assunção Ferreira

**Affiliations:** I Universidade Federal do Rio de Janeiro. Rio de Janeiro, Rio de Janeiro, Brasil

**Keywords:** Fibromialgia, Diagnóstico, Terapêutica, Enfermagem, Representação Social

## Abstract

**Objetivo:**

Analisar as representações sociais da fibromialgia baseadas em seus sintomas e suas influências no diagnóstico e na terapêutica.

**Métodos:**

Pesquisa qualitativa com aplicação da Teoria das Representações Sociais e método de amostragem *snowball*. Realizaram-se entrevistas semiestruturadas com 30 pessoas adultas, diagnosticadas com fibromialgia, na cidade do Rio de Janeiro, Brasil, entre abril de 2020 e janeiro de 2021. Foi realizada análise estatística e lexicográfica pelo *software Alceste*.

**Resultados:**

A dor, como fenômeno subjetivo, dificulta sua legitimidade, o diagnóstico e a terapêutica, potencializando o sofrimento. Informações insuficientes geram julgamentos, estereótipos e preconceitos.

**Considerações finais:**

Estigmas, preconceitos, a variedade e a invisibilidade sintomatológica dificultam a objetivação da doença nos moldes cartesianos-biomédicos, gerando peregrinação diagnóstica, equívocos e desafios no tratamento. Tais representações interditam as relações e o manejo da doença. Desconstruí-las é um caminho para melhor cuidar dos que têm fibromialgia. Sensibilizar as pessoas e difundir informações qualificadas são importantes aliados.

## INTRODUÇÃO

A fibromialgia é uma doença crônica reumática, caracterizada por dor difusa, com duração maior que três meses, fadiga, alterações cognitivas e funcionais, distúrbio do sono, disfunções vesicointestinais e, geralmente, associada à ansiedade e depressão^(1, 2, 3)^.

Sua etiopatogenia é complexa e ainda não completamente esclarecida, assim como sua predominância no sexo feminino, numa proporção de até nove mulheres, frequentemente na faixa etária de 35 a 44 anos, para cada homem^(4, 5)^. Um estudo de revisão evidencia a prevalência da fibromialgia no gênero feminino e aponta que isso pode ser um fator de risco para a doença^(6)^.

A fibromialgia afeta 4,2% das mulheres entre 40 e 50 anos em comparação com 0,2% dos homens, sendo que estas mulheres têm baixo nível de escolaridade e vivem em estado de vulnerabilidade social, política e econômica^(5, 7, 8)^. Além disso, mulheres com menos de 60,6 anos tendem a apresentar sintomas mais agudos da doença^(7)^. As mulheres são 1,5 vezes mais susceptíveis a sentir dor crônica generalizada do que os homens e possuem 10 vezes mais chances de ter 11 ou mais *tender points* no exame clínico, fato este que pode explicar a prevalência maior de fibromialgia em mulheres^(9)^.

Globalmente, a incidência da fibromialgia varia entre 0,2% e 8%³. Estima-se que acometa de 2% a 4% da população mundial e até 2,5% da população brasileira^(4, 5)^. Contudo, devido à grande heterogeneidade sintomatológica e à ausência de procedimentos diagnósticos padronizados, esses dados podem sofrer variações e repercutir na condução do tratamento^(10)^.

O diagnóstico da fibromialgia é predominantemente clínico e examinador-dependente, realizado com base na avaliação da presença de dor e sensibilidade nos *tender points*, associados ao julgamento clínico dos sinais e sintomas referidos pelos indivíduos. Tal fato explica as inúmeras variações diagnósticas de acordo com a experiência do médico, não havendo um marcador laboratorial ou um exame de imagem que comprove sua existência^(4, 9)^.

Para tentar mitigar essas disparidades diagnósticas, o Colégio Americano de Reumatologia (ACR) elaborou, em 1990, critérios para nortear a avaliação clínica e diagnóstica de pessoas com fibromialgia. Porém, esses critérios supervalorizavam a identificação dos pontos dolorosos em detrimento dos demais sintomas. Mais tarde, em 2010, o ACR reformulou esses critérios, apontando a importância da história clínica, a avaliação da dor generalizada e da gravidade dos sintomas para o diagnóstico. Em 2017, foi lançado um documento com novas diretrizes, onde especialistas recomendaram a aplicação dos critérios diagnósticos de 2010 e sugeriram a avaliação dos *tender points* em conjunto com a identificação de outros distúrbios funcionais apresentados, como a dor difusa, fadiga, distúrbios do sono e alterações cognitivas^(4, 11)^. Percebe-se através desse percurso, o quanto ainda há divergências diagnósticas e a amplitude desse desafio diante da não especificidade e subjetividade dos sintomas.

Sendo assim, estudos indicam que possa haver superdiagnóstico de fibromialgia em mulheres e subdiagnóstico em homens, causando imprecisão nas estatísticas relacionadas a sintomas, prevalência, custos, comorbidade e resultados clínicos. No entanto, estes mesmos estudos sugerem que existe um importante elemento de construção social sobre a fibromialgia e em sua identificação, evidenciando as dimensões médicas e sociais desta enfermidade^(12, 13)^.

Toda essa ampla variação nas manifestações clínicas da fibromialgia sofre influência dos aspectos sociais, psicológicos e culturais^(14)^. Portanto, pode-se afirmar que a fibromialgia, além de suas variáveis biológicas e somáticas, também envolve os aspectos psicossociais que perpassam por todo o processo saúde/doença, afetando negativamente o físico, cognitivo, social, familiar e profissional das pessoas adoecidas^(7)^.

Nesse sentido, questiona-se: como se expressam os sintomas da fibromialgia nas representações sociais de pessoas com esta doença e quais são as influências no seu diagnóstico e terapêutica?

Justifica-se buscar resposta a esta questão de pesquisa porque pessoas com fibromialgia percebem a doença como um distúrbio estigmatizado, invisível e de difícil compreensão^(15)^, o que agrava o sofrimento de quem convive com esta enfermidade gerando respostas humanas que podem ser mais bem manejadas pela enfermagem, quando conhecidas.

Aspectos psicossociais que envolvem fenômenos de saúde e doença indicam a confluência entre a psicologia e a sociologia na sua compreensão e uma ferramenta potente para estudar tais fenômenos é pela via das representações sociais^(16, 17)^. Representações sociais são formas de conhecimento prático que estabelecem elos entre o saber e o fazer, formadas por processos de concretização do que é abstrato, não familiar, chamado de objetivação, e por aproximações com quadros de referências sobre o que já é previamente conhecido ou familiar, chamado de ancoragem. Logo, conhecer as representações sociais implica em desvelar os seus processos construtores, quando se aplica a abordagem socioantropológica^(17)^.

## OBJETIVO

Analisar as representações sociais da fibromialgia baseadas em seus sintomas e suas influências no diagnóstico e na terapêutica.

## MÉTODOS

### Aspectos éticos

O projeto foi submetido ao Comitê de Ética em Pesquisa da Escola de Enfermagem Anna Nery e ao Instituto de Saúde São Francisco de Assis da UFRJ, sendo aprovado em 16 de março de 2020. Na ocasião do convite para participação na pesquisa, a pesquisadora principal se apresentou às pessoas contatadas, bem como informou sobre a pesquisa, e todos os participantes assinaram o termo de consentimento livre e esclarecido ao aceitarem o convite.

### Referencial teórico-metodológico

Aplicou-se o referencial da Teoria das Representações Sociais, considerando os conceitos de objetivação e ancoragem na formação de representações sociais^(16, 17)^.

### Tipo de estudo

Trata-se de um estudo qualitativo, descritivo e exploratório, no qual foram aplicadas as diretrizes do *Consolidated Criteria for Reporting Qualitative Research* (COREQ).

### Procedimentos metodológicos

Os participantes foram recrutados pelo método snowball sampling, utilizado quando há dificuldades em acessar a população-alvo do estudo ou quando a amostragem probabilística inicial é impraticável^(18)^. O alcance dos participantes teve início a partir de duas “sementes”, mulheres com diagnóstico de fibromialgia residentes no estado do Rio de Janeiro, indicadas por uma pessoa da rede de relações da pesquisadora principal. Por meio dessas duas indicações (sementes), foram recrutados novos participantes com o perfil do estudo, sendo todos contatados por telefone. Com base na recomendação de consenso para amostras qualitativas e no critério de saturação para suficiência dos dados^(19)^, a coleta de dados foi encerrada após alcançar 30 participantes. Todas as pessoas contatadas concordaram em participar da pesquisa, e não houve desistências durante o processo.

### Fonte de dados

Os critérios de inclusão dos participantes foram ter 18 anos ou mais e possuir diagnóstico de fibromialgia. Os critérios de exclusão incluíram pessoas com comprometimento cognitivo ou de fala que dificultasse a coleta de dados, bem como adolescentes e/ou jovens com diagnóstico de Síndrome da Fibromialgia Juvenil, pois essa variável requer uma análise diferenciada sobre as repercussões da doença no cotidiano.

### Coleta e organização dos dados

A coleta foi realizada pela pesquisadora principal no período de abril de 2020 a janeiro de 2021, por meio de entrevistas individuais presenciais, com gravação de voz, com duração entre 60 e 90 minutos. Os protocolos de segurança vigentes durante o distanciamento social devido à covid-19 foram seguidos. As entrevistas ocorreram nas residências dos participantes, conforme sua livre escolha.

Foram utilizados dois instrumentos: o primeiro continha perguntas para levantamento de dados pessoais e laborais (como sexo, idade, estado conjugal, ocupação), histórico de saúde-doença, história familiar, clínica e medicamentosa, sintomas, acompanhamento de saúde (rede privada ou pública), uso de métodos de tratamento não farmacológicos, participação em grupos de apoio e frequência. O segundo instrumento consistiu em perguntas abertas, apresentadas por meio de um roteiro com questões semiestruturadas, explorando quem sabe, de onde sabe, o que e como sabe sobre o fenômeno, sobre o que e seu efeito^(17)^. Ou seja, foi realizada uma exploração dos saberes e das práticas sobre o que está sendo pesquisado. Nesse sentido, as perguntas abordaram a doença e suas manifestações clínicas, a descoberta e os desafios do diagnóstico e do tratamento da fibromialgia, as relações com pessoas que têm e não têm a doença, as práticas de cuidado e seus aprendizados, as dificuldades, as redes de apoio e o que é necessário saber mais sobre a doença e sobre os cuidados.

### Análise dos dados

Os dados do perfil dos participantes foram tratados estatisticamente, aplicando-se a frequência absoluta e relativa. Os textos das entrevistas formaram um corpus de 30 unidades de contexto inicial (UCI) após transcrição, correspondente ao número de entrevistas processadas pelo *software Alceste*. Este *software* realiza análise lexical contextual de um conjunto de segmentos de texto e aplica cálculos sobre a coocorrência de palavras nesses segmentos para definir classes de palavras que representem diferentes discursos sobre o objeto da investigação. Dessa forma, identifica oposições lexicais e alcança as oposições de diferentes pontos de vista coletivos expressos no vocabulário de um texto, permitindo acessar a comunicação intergrupos, a partilha de conhecimentos e a produção de representações sociais^(20)^.

O processamento dos textos no software promove o agrupamento das raízes semânticas a partir das palavras empregadas pelos participantes nos discursos para comunicar seus pontos de vista sobre o objeto investigado. A partir deste agrupamento, o *software* gera figuras (dendrogramas), como a Classificação Hierárquica Descendente (CHD), que evidencia as palavras mais significativas da classe por meio de associação estatística expressa no valor de *Phi*. A CHD é composta pelas formas reduzidas das palavras a partir de seus radicais, e as formas completas (as palavras) são listadas no corpo do relatório gerado pelo *software*, juntamente com as respectivas quantidades de aparição nas classes lexicais e o valor estatístico de associação à classe (valor de *Phi*). Para compreender o motivo da aparição das palavras, recorre-se aos fragmentos de texto que compõem cada classe, expressando as ideias que lhe dão sentido, chamadas de Unidades de Contexto Elementar (UCE).

Para atender ao objetivo proposto, este artigo trata das duas classes formadas a partir dos léxicos que abordam os sintomas, a peregrinação diagnóstica e a terapêutica da fibromialgia. A Classe 2, nomeada como “Uma dor que anda: a fibromialgia e seus sintomas”, e a Classe 3, nomeada como “Desafios do diagnóstico: um caminho de peregrinação”, pois essas classes estão relacionadas com a vasta sintomatologia da doença e os desafios diagnósticos devido à sua não especificidade.

## RESULTADOS

Com relação ao perfil dos 30 participantes, houve um predomínio do sexo feminino em 93% (n=28). Quanto à faixa etária, 67% têm entre 41 e 60 anos (n=20), seguidos de 30% com 20 a 40 anos (n=9) e 3% com 61 anos ou mais (n=1). Ainda, 63% são casados (n=19) e 70% têm ocupação laboral (n=21). Com relação ao tempo de diagnóstico da fibromialgia, 40% o receberam há 10 anos ou mais (n=12). O acompanhamento de saúde se dá majoritariamente na rede privada, por 93% dos participantes (n=28). Quanto ao tratamento, 97% (n=29) utilizam fármacos e 3% (n=1) não os utilizam; 53% não seguiam tratamentos não farmacológicos (n=16) e 47% faziam uso de algum tipo de tratamento não farmacológico (n=14). A participação em grupos de apoio é feita por 40% (n=12), e todos estes participavam de grupos virtuais em redes sociais como *Facebook, WhatsApp* e canais do *Youtube*.

Quanto aos dados das entrevistas, o software subdividiu o texto em 4.034 unidades de contexto elementar (UCE), formadas por 8.138 formas distintas. O software reduziu essas palavras em raízes distintas, compondo 1.287 palavras analisáveis e 309 palavras suplementares. Foram analisadas 2.934 UCE, resultando em 73% de aproveitamento do corpus.

A classe 2 foi formada por 370 UCE e 112 palavras analisáveis, correspondendo a 13% do corpus classificado para análise. Ao analisar a CHD dessa classe, a forma “Pern” teve o maior valor de Phi, correspondendo a 0,32. Esse radical se expressa nas formas completas “perna”, “pernas”. Em seguida, aparecem os termos “sono”, “dorm”, “dor”, “doi”, conforme constam no Quadro 1.

**Quadro 1:** Palavras representativas da Classe 2, conforme o dendrograma da Classificação Hierárquica Descendente (CHD), Rio de Janeiro, Brasil, 2021

CLASSE 2 – 370 Unidades de Contexto Elementares		
Forma reduzida	Forma completa	Valor de *Phi*
pern	Perna (35) pernas (47)	0,32
sono	Sono (48)	0,26
dorm	Dorme (5) dormência (6) dormente (6) dormia (5) dormir (51) dormiu (1)	0,23
dor	Dor (295) dores (68)	0,23
doi	Dói (49) doía (15)	0,22
nas	Nas (64)	0,21
durmo	Durmo (24)	0,19
braço	Braço (12) braços (17)	0,17
joelho	Joelho (28) joelhos (4)	0,17
acord	Acorda (2) acordada (3) acordar (3) acordava (9) acordei (4) acordo (13)	0,16
pescoço	Pescoço (15)	0,16
cost	Costas (22) costela (3) costelas (2)	0,16
noite	Noite (14) noites (2)	0,15
insônia	Insônia (15)	0,15
sensibilida	Sensibilidade (13)	0,15
and	Anda (7) andando (4) andar (18) andava (5) andei (4) ando (3)	0,14
sint	Sinto (78)	0,13
amanhec	Amanhece (2) amanhecer (1) amanhecia (3) amanheço (4)	0,13
musculatu	Musculatura (9)	0,13
mão	Mão (13) mãos (16)	0,13
parec	Parece (34) parecia (12)	0,12
forte	Forte (16) fortes (7)	0,12
inch	Incha (2) inchação (1) inchada (2) inchado (2) inchando (4) inchar (1) inchava (1)	0,12
semana	Semana (26) semanas (2)	0,12
musculos	Músculos (6)	0,11
**Forma reduzida Forma completa Valor de *Phi***		
pé	Pé (24)	0,11
cansaço	Cansaço (17)	0,11

Nomeada como “Uma dor que anda: a fibromialgia e seus sintomas”, a Classe 2 evidencia os léxicos que se associam à apresentação sintomatológica da doença e como, quando e onde essas pessoas sentem esses incômodos. Identifica-se nos resultados que há uma tentativa de identificar os sintomas apresentados pela doença, bem como sua localização, duração e intensidade, como uma estratégia observacional de compreender como a doença se manifesta, como é percebida, caracterizada e quais situações e comportamentos as potencializam ou minimizam.

*Dor, muita dor, dói mais as articulações na parte superior, dedo, as minhas articulações todas, braços e as costas. Ultimamente eu não sentia a dor muito nas pernas, agora eu venho sentindo muito*. (UCI 24, mulher, 41-60 anos)

*Sintomas são dores e cansaço. Desânimo, você nunca descansa. E o corpo. É como se você nunca tivesse descanso mesmo. A sensação que dá é que você faz trabalho muito pesado, trabalho braçal vinte e quatro horas. Então, o médico me disse que a questão de sono, questão de humor, tudo estaria relacionado a fibromialgia*. (UCI 9, mulher, 41-60 anos)

*É diferente de eu estar carregando a mochila pesada, é diferente, o peso vai estar em um lugar só, específico, naquele dia. Vai estar nas minhas pernas, então eu vou estar com uma dor insuportável nas minhas pernas, ou no pescoço. Quantas vezes eu já chorei pedindo a Deus arranca meu pescoço, arranca minhas pernas, porque é uma dor insuportável quando eu estou em crise*. (UCI 6, mulher, 20-40 anos)

*Eu apelidei por isso, é uma dor móvel, não é uma dor permanente em um mesmo lugar não. E quando dá a crise, dói e dói muito. Eu passei uma fase que me dava dor nesse dedinho aqui. O que eu pegava na mão, caía. Tinha que levantar para pegar as coisas e doía, chegava a dar aquele choque*. (UCI 10, mulher, 41-60 anos)

*Então assim, eu estava com uma dor no braço inicialmente, mas depois de dois dias essa dor já estava no meu tórax e pescoço. E aí eu tinha que observar como que isso aconteceu, com quanto tempo que essa dor foi para outro lugar do corpo, aonde que estava doendo mais*. (UCI 17, mulher, 20-40 anos)

*Porque eu sinto dor direto, entendeu?! Eu já observei assim, nas articulações doem muito, mas eu já observei que quando eu fico nervosa por algum motivo, ou ansiosa por alguma coisa, a dor vem intensa, ela é mais intensa, entendeu?!* (UCI 19, mulher, 41-60 anos).

A classe 3, por sua vez, foi formada por 637 UCE e 129 palavras analisáveis, correspondendo a 22% do corpus classificado para análise. Ao analisar a CHD dessa classe, a forma “Exame” teve o maior valor de Phi, correspondendo a 0,32. Esse radical se expressa nas formas completas “exame”, “exames”. Em seguida, aparecem os termos “reumatolog”, “tom”, “med”, “remed”, conforme constam no Quadro 2.

**Quadro 2:** Palavras representativas da Classe 3, conforme o dendrograma da Classificação Hierárquica Descendente (CHD), Rio de Janeiro, Brasil, 2021

CLASSE 3 – 637 Unidades de Contexto Elementares		
Forma reduzida	Forma completa	Valor de Phi
exame	Exame (62) exames (80)	0,32
reumatolog	Reumatológica (1) reumatológicas (1) reumatológico (2) reumatologista (89)	0,27
tom	Toma (15) tomado (5) tomando (47) tomar (72) tomasse (2) tomava (19) tome (1)	0,26
med	Mede (1) media (2) médica (15) medicar (1) médico (153) médicos (21) medir (3)	0,23
remed	Remedinho (1) remédio (82) remédios (31)	0,21
medica	Medicamento (60) medicamentos (28)	0,20
ortoped	Ortopedia (3) ortopédica (1) ortopedista (37) ortopedistas (5)	0,20
consult	Consulta (19) consultar (9) consultas (3) consultava (1) consultei (6) consulto (1)	0,18
fiz	Fiz (79) fizer (3) fizeram (6) fizesse (5)	0,17
psiquiatr	Psiquiatra (49) psiquiátrica (1)	0,17
os	Os (150)	0,16
sangue	Sangue (26)	0,16
velija	Velija (32)	0,16
tratament	Tratamento (60) tratamentos (4)	0,16
doutor	Doutor (26) doutora (13)	0,15
itaperuna	Itaperuna (27)	0,15
ano	Ano (43) anos (108)	0,15
diagnostic	Diagnosticada (7) diagnosticado (4) diagnosticar (5) diagnosticaram (2)	0,14
neurolog	Neurológico (1) neurologista (24) neurologistas (1)	0,14
fiqu	Fique (1) fiquei (72)	0,14
mes	Mês (12) mesa (1) meses (37)	0,13
uns	Uns (81)	0,13
marc	Marca (3) marcada (2) marcar (10) marco (8) marcou (3)	0,13
fez	Fez (50)	0,13
trat	Trata (2) tratado (1) tratando (13) tratar (22) trataram (2) tratava (2) tratei (6)	0,13
nel	Nela (3) nele (24)	0,13
intern	Interna (1) internada (4) internado (6) internar (6) internaram (1) internasse (1)	0,13

Essa classe possui aproximação com o percurso diagnóstico da doença, bem como as condutas terapêuticas atreladas a ela.

A Classe 3 foi nomeada “Desafios do diagnóstico: um caminho de peregrinação”. As UCE dessa classe estão relacionadas ao processo diagnóstico e terapêutico da fibromialgia. Por meio dos léxicos, compreende-se o percurso de busca pelo nome da dor, em seus aspectos cronológicos e de entendimento, e uma forma capaz de saná-la. Os resultados mostram que as pessoas com fibromialgia enfrentam um longo caminho em busca do diagnóstico, muitas vezes levando-os a se indagarem se realmente têm alguma doença, colocando em dúvida o que sentem e a proporção com que sentem.

Por ser um diagnóstico clínico, a fibromialgia torna-se, muitas vezes, um diagnóstico de exclusão, devido à ausência de exames e marcadores laboratoriais que a confirmem. Além disso, muitas vezes a doença está associada a outros diagnósticos, o que pode contribuir para o aumento dos subdiagnósticos. Essa peregrinação maximiza os sintomas apresentados por essas pessoas e potencializa quadros depressivos e ansiosos.

*E fiquei dez dias internado. Fizeram tudo quanto é exame: tomografia, eletrocardiograma, ressonância, exame de sangue, tudo quanto é exame eles fizeram, e não acusava nada, né?! Aí eu passei do hospital, eu passei para um neurologista, para um cardiologista. Depois foi para um reumatologista. O reumatologista que descobriu.* (UCI 20, homem, 20-40 anos)

*Eu comecei a tomar o velija* [medicamento] *e melhorei muito, muito, muito. Voltei nele, ele viu os exames e falou assim: realmente fulana, você não tem artrite reumatoide. Seu diagnóstico é de fibromialgia. E aí foi uma libertação, porque aí você sai daquela loucura, o que você tem, porque você não melhora.* (UCI 21, mulher, 41-60 anos)

*E foi daí que eu descobri e comecei fazendo o tratamento. Ah, uns oito meses, uns oito meses sentindo dor. Inclusive, nesses oito meses eu ia para o pronto atendimento, chegava lá com muita dor e eles queriam fazer morfina de qualquer maneira.* (UCI 17, mulher, 20-40 anos)

*Eu não sei o que é aquilo que o índio vende. Eu não sei nem o que é aquilo, mas aquilo que eu tomo é o que me alivia. Não, não tem nome, não tem nada. Vem assim, dez envelopes de papel alumínio, que vou te mostrar, dobrado desse tamanho, com um pó branco dentro.* (UCI 26, mulher, 41-60 anos)

*Todo mundo que eu conheci pessoalmente, inclusive daqui, foi para o fundo do poço com depressão. Eu já fiquei, vou te falar que eu não tive uma síndrome do pânico, um negócio assim não, mas eu fiquei, passei já uns meses, lá no quarto.* (UCI 5, mulher, 41-60 anos)

## DISCUSSÃO

O perfil dos participantes reitera o que se conhece sobre o grupo acometido pela fibromialgia, que é composto majoritariamente por mulheres e por pessoas na idade adulta. Contudo, a escassez de estudos com amostras robustas de homens dificulta análises aprofundadas e apropriadas sobre as especificidades da doença em relação à variável gênero^(6)^.

Sobre as características clínicas, a fibromialgia é conhecida por sua variedade sintomatológica, como demonstrado pelos dados da Classe 2. Contudo, dentre os sinais e sintomas mais apontados por quem vive com essa doença, a dor é o sintoma central, com alterações apenas no lugar onde dói. Esse dado vai ao encontro de outros estudos que afirmam que a dor é o sintoma mais expressivo, recorrente e impactante para as pessoas com fibromialgia^(21, 22, 23)^.

Esta é caracterizada pela presença de dor crônica, com duração maior que três meses, com palpação dolorosa de pontos específicos, denominados tender points, e ausência de processo inflamatório. Geralmente, há uma forte relação entre o número identificado de tender points e a intensidade de estresse e depressão, muitas vezes justificada pelos danos emocionais comumente presentes em quem convive com a dor crônica^(4, 22, 23)^.

Outros sintomas também são muito citados por quem tem fibromialgia, como sono não reparador, cansaço excessivo, desânimo, alterações do humor, da concentração e da memória, rigidez e/ou fraqueza muscular, dormência, síndrome do cólon irritável, bexiga irritável, cefaleia, vertigem, confusão mental, xerostomia, xeroftalmia, sensibilidade ao frio, entre outros^(24, 25, 26)^.

Contudo, a dor é a principal queixa para as pessoas com fibromialgia, e esta é uma percepção sensorial muito particular. Muitas vezes essas pessoas tentam caracterizá-la, objetivá-la e nominá-la para que seja possível falar sobre ela^(17)^. Dentre as características apontadas, destaca-se a dor móvel, a dor que anda, a dor em choque, em pontadas, em queimação, a dor intensa que paralisa, que enjoa, que provoca hipersensibilidade, a dor cruel e insuportável. Todas essas caracterizações são tentativas de comunicar o que é a fibromialgia para quem não a sente em seu corpo, para objetivá-la dando concretude a algo que é sub-jetivo^(16)^. Nominá-la como uma “dor móvel, uma dor que anda” é uma metáfora que traduz a representação de uma sensação dolorosa que percorre o corpo, que está a cada vez em um lugar distinto, o que leva a pessoa a ter múltiplas queixas.

Pessoas com fibromialgia buscam ancorá-la em algum saber pré-existente, uma experiência ou dor já sentida, e a caracterizam aproximando-a de uma sensação dolorosa anormal, a exemplo da dor em choque, em pontadas e em queimação. Nota-se que há uma variedade de percepções acerca da experiência dolorosa e uma tentativa de caracterizá-la a partir da observação, procurando entender seu comportamento, suas particularidades e como a mesma se manifesta na pessoa^(21)^.

Portanto, para conseguir caracterizá-la e torná-la compreensível para os outros, as pessoas com fibromialgia entendem que é preciso observação. Através da observação percebem as semelhanças e diferenças da experiência dolorosa em situações variadas, como na condição de oscilação de temperatura ambiente; compreendem a temporalidade da dor, presente desde a infância e carregada de concepções emotivas; a frequência e o posicionamento da dor, sendo esta constante ou intermitente, fixa ou móvel; os aspectos e condições que a maximizam, como o estresse e ansiedade, ou a minimizam, como a implementação de um tratamento farmacológico ou não, entre outras percepções.

Sendo assim, por ter um sintoma central subjetivo e multi-fatorial, que afeta o somático e o social, para entender a fibromialgia é preciso uma observação atenta de como a doença e seus sintomas se comportam, como estes se apresentam, suas características, seus impactos nas inúmeras dimensões da vida, bem como quais estratégias de cuidado, farmacológicas e não farmacológicas são eficazes para a sua redução. A busca por entendê-la é primordial para direcionar a ação a ser adotada. Nesse sentido, pensamento, saber e ação se cruzam na tomada de decisão sobre como agir em relação ao fenômeno^(17)^, no caso a fibromialgia. E aí se identifica o movimento da representação social sobre a fibromialgia: desenvolver um saber sobre ela para aprender a falar sobre a sua doença, lidar com ela e agir sobre ela^(16)^, em termos de cuidados.

Devido a essa vasta sintomatologia e subjetividade, a fibromialgia torna-se um grande desafio diagnóstico, ocasionando uma verdadeira peregrinação entre médicos e especialistas em busca de um nome para a dor, conforme evidenciado pela Classe 3.

Além disso, trata-se de uma síndrome de causa ainda desconhecida, rotulada como uma doença “medicamente inexplicável”, pois não é confirmada através de exames e procedimentos diagnósticos tradicionais, mas sim por ter critérios diagnósticos subjetivos não associados a alterações físicas^(24, 27)^. Desse modo, até que a doença seja diagnosticada, as representações sobre a fibromialgia a põem em suspeição como uma enfermidade não explicada e obscura, se fazendo presentes nos discursos médicos e leigos, contribuindo com a hegemonia do discurso biomédico cartesiano.

Pessoas com fibromialgia referem muitas dificuldades em receber um diagnóstico correto, fato que se relaciona com a complexidade na sua validação e no próprio desconhecimento e incompreensão dos profissionais médicos acerca dos critérios diagnósticos e como avaliá-los^(28, 29)^.

Por ter esse caráter subjetivo, a fibromialgia é muitas vezes considerada uma “doença invisível” e esta denominação também retrata uma representação que influencia na demora do diagnóstico e do tratamento. Essa invisibilidade tende a gerar desarranjos nos papéis e responsabilidades pessoais, sociais, laborais e familiares, além de ocasionar uma peregrinação diagnóstica que nem sempre evidencia alguma anormalidade ou alteração que justifique suas dores e demais sintomas^(27)^. Em média, pessoas com fibromialgia chegam a passar entre 4 a 10 anos aguardando o diagnóstico correto^(21, 30)^.

Essa peregrinação em busca do diagnóstico, com inúmeras consultas médicas e exames, gera frustração e desânimo. Trata-se de uma busca pela legitimação da dor que se sente, onde algumas pessoas passam anos sem saber ao certo o que têm e qual é o real diagnóstico. Esse percurso também envolve tratamentos incorretos, além de gerar descrédito e falta de esperança em relação ao prognóstico.

Sendo assim, viver com fibromialgia, com toda sua subjetividade e caráter sindrômico, leva à experiência da estigmatização. Geralmente, pessoas com fibromialgia são incompreendidas em sua dor, e por isso são rejeitadas e julgadas como preguiçosas. Esta estereotipia reforça e é reforçada pelas representações da fibromialgia como uma doença invisível. Representações comunicam imagens e ideias sobre coisas e pessoas, veiculam informações, crenças, preconceitos, julgamentos e, em vista disso, geram comportamentos e atitudes, e todo o estigma da doença fibromialgia recai sobre o doente, por meio de denominações que o enquadram em imagens previamente definidas com efeitos sociais^(31)^, por isso o diagnóstico é interpretado como uma libertação, como consta na UCI 21.

Por não apresentar um sinal visível, algo palpável que justifique a dor, o diagnóstico também se torna majoritariamente clínico e diferencial, como também demorado, que depende da habilidade clínica do médico que avalia^(27, 32, 33)^. Este dado reforça a necessidade de investimentos na formação profissional médica para aplicar critérios precisos de diagnóstico da fibromialgia, como já apontaram estudos sobre o tema^(28, 29)^.

Quando realizada de forma correta e abrangente, respeitando e valorizando a queixa do paciente, a abordagem diagnóstica tende a minimizar a jornada dessas pessoas em inúmeras consultas, reduzindo os gastos com recursos de saúde, o consumo excessivo de medidas farmacológicas e não farmacológicas sem real eficácia, prevenindo, portanto, complicações e hospitalizações^(30, 32)^. Sem contar que, ao ter conhecimento acerca da doença que se tem, que a mesma é real, tal fato reduz consideravelmente o sofrimento e potencializa as perspectivas de melhora do quadro^(32)^.

Com relação às condutas terapêuticas, o tratamento farmacológico é amplamente implementado para as pessoas com fibromialgia, embora não haja um protocolo específico^(33)^. Em contrapartida, é possível identificar uma baixa adesão na continuidade do tratamento medicamentoso, pois estes não promovem a cura e possuem efeitos adversos indesejáveis^(1)^. Os tratamentos farmacológicos são de longo prazo e não geram respostas efetivas na minimização da dor, além dos efeitos colaterais que são diversos; por essa razão, cada vez mais cresce o interesse pela adoção de práticas não farmacológicas, complementares ao tratamento da fibromialgia^(34)^. Isto encontra respaldo nos dados de perfil dos participantes, em que majoritariamente (97%) usam fármacos e quase metade (47%) fazem uso de algum método não farmacológico.

Tratamentos não farmacológicos como a prática de exercício físico, o pilates, a fisioterapia, a acupuntura, a psicoterapia, também são amplamente recomendados para pessoas com fibromialgia. Os exercícios físicos em pessoas com fibromialgia reduzem a dor, melhoram a qualidade de vida em relação à saúde e têm efeito benéfico em quadros de depressão^(35)^. A estimulação elétrica e a eletroacupuntura também têm sido objeto de pesquisas para casos de fibromialgia, a exemplo de uma pesquisa de meta-análise que indicou baixa qualidade de evidências para a eficácia da estimulação elétrica e evidências de moderada qualidade para a eletroacupuntura para alívio da dor^(36)^. Um tratamento não farmacológico que também pode ser citado é o da Terapia Comunitária Integrativa, que se mostrou como uma estratégia potente para construir e ampliar conhecimentos sobre a fibromialgia e no empoderamento dos participantes para o autocuidado^(37)^.

Na maioria das vezes, as condutas não farmacológicas são associadas aos fármacos comumente prescritos como alternativas para a potencialização dos resultados na redução da dor e outros sintomas. Nem sempre todas as pessoas com fibromialgia se beneficiam e alcançam bons resultados com tais terapêuticas. As terapêuticas disponíveis desempenharão efeitos individualizados; portanto, cada pessoa observará determinados resultados e perceberá qual conduta terapêutica é a mais adequada à sua condição.

Contudo, o que se percebe é a busca por uma alternativa que proporcione o alívio para a dor, seja qual for essa alternativa, qual sua origem e composição, quais os possíveis efeitos adversos. A busca de alívio leva as pessoas a usarem produtos que nem sempre sabem exatamente o que é, aderem a uma crença de que aquela determinada alternativa gera o efeito procurado, já que outras terapêuticas não foram satisfatórias, a exemplo do uso de “um pó branco fornecido por um índio”, conforme consta na UCE 26.

Outro fator complicador para o diagnóstico é a associação da fibromialgia a outras patologias. Dentre as doenças associadas ao diagnóstico de fibromialgia, pode-se citar a depressão, a ansiedade, doenças osteomusculares e articulares, doenças autoimunes, como a artrite reumatoide e a artrite psoriática, hipertensão arterial sistêmica, entre outras. A coexistência entre fibromialgia e outras condições patológicas pode dificultar a avaliação, o diagnóstico e o tratamento devido a uma interpretação inadequada da apresentação clínica da doença^(30)^.

Desse modo, reafirma-se a importância do acompanhamento multidisciplinar e do olhar integral às pessoas com fibromialgia, para além da dor. A fragmentação proposta pelo modelo biomédico não atende a contento a saúde de um indivíduo e seu completo bem-estar. Há um maior retorno às necessidades da pessoa e uma maior resolutividade de seus problemas quando o foco da atenção em saúde está voltado para sua totalidade^(38)^.

Neste intento, conhecer os saberes e as práticas de cuidado e de autocuidado das pessoas com fibromialgia pode auxiliar o profissional de saúde, em especial a enfermeira, a buscar cuidados que atendam mais amiúde às necessidades de quem sofre com esta enfermidade. Por isso, as pesquisas de representações sociais são aplicadas na saúde, pois representações são conhecimentos práticos, cujas dimensões se compõem de informações, atitudes (práticas) e campo, que é onde a representação se organiza com base em seus elementos explicativos^(16, 17)^. Ao desvendá-las, os profissionais podem planejar ações de educação em saúde que desmistifiquem estereótipos e auxiliem as pessoas nas escolhas de cuidados que as ajudem em seus processos de tratamento.

### Limitações do estudo

Este estudo apresenta algumas limitações que devem ser consideradas ao interpretar os resultados. A primeira refere-se ao fato de a pesquisa ter sido realizada durante o período da pandemia de covid-19, e as reuniões de grupos de apoio de pessoas com fibromialgia foram suspensas, e a captação dos participantes foi feita pela técnica de amostragem bola de neve, o que dificultou a obtenção de uma amostra com características mais diversificadas. Nesse sentido, não foi possível ampliar as análises para possibilitar a identificação de convergências e divergências de representações por gênero e faixa etária.

### Contribuições para a área da Enfermagem, Saúde ou Política Pública

Conhecer as representações sociais de doenças, em especial quando são produtoras de estigmas e preconceitos, possibilita à enfermagem acessar elementos psicossociais que geram sofrimento e influenciam no cuidado aos pacientes. Com esse conhecimento, potencialmente, a oferta de cuidado tende a ser mais específica e direcionada ao que os pacientes necessitam para auxiliá-los, bem como às suas famílias e redes de apoio, para ajudá-los a desconstruir representações que interditam as relações e o bom manejo da doença e do sofrimento por ela gerado. Ampliam-se, assim, os saberes, os cuidados e as estratégias para melhor lidar com a doença e o paciente em seus contextos de vida, por meio de informações qualificadas sobre a doença.

## CONSIDERAÇÕES FINAIS

A fibromialgia tem uma apresentação sindrômica, com sintomas inespecíficos e subjetivos, sendo a dor o principal deles. Essa variedade e invisibilidade sintomatológica, sem causa conhecida ou aparente, dificultam a objetivação da doença de acordo com o modelo cartesiano-biomédico, reforçam representações como doença invisível e geram diferentes nominações sobre a dor pelos pacientes, na tentativa de caracterizá-la e torná-la objetiva para o outro. Traduzir a fibromialgia como uma dor móvel, uma “dor que anda” e, portanto, se desloca pelo corpo, mostra o esforço cognitivo dos pacientes em explicar a dificuldade de localizar a doença no corpo, como é próprio do modelo biomédico. Tudo isso culmina em uma peregrinação diagnóstica em busca da libertação, condição que representa o tão esperado diagnóstico que, até que ocorra, leva a inúmeros equívocos e desafios na condução terapêutica da fibromialgia. Portanto, o aprimoramento do diagnóstico mostra-se uma necessidade urgente na formação médica.

Representações se formam com base em três dimensões, sendo duas delas a informação e a atitude. A difusão de informação qualificada sobre a fibromialgia se apresenta como um bom caminho para a sensibilização dos familiares e da sociedade em geral em relação à doença, ajudando na sua melhor compreensão, na desconstrução de estereótipos e preconceitos para que as pessoas que sofrem com fibromialgia possam ser mais bem compreendidas, acolhidas e melhor cuidadas.
